# Examining Self-Weighing Behaviors and Associated Features and Treatment Outcomes in Patients with Binge-Eating Disorder and Obesity with and without Food Addiction

**DOI:** 10.3390/nu13010029

**Published:** 2020-12-23

**Authors:** Ashley A. Wiedemann, Valentina Ivezaj, Ralitza Gueorguieva, Marc N. Potenza, Carlos M. Grilo

**Affiliations:** 1 Department of Psychiatry, Yale School of Medicine, New Haven, CT 06511, USA; ashley.wiedemann@yale.edu (A.A.W.); valentina.ivezaj@yale.edu (V.I.); carlos.grilo@yale.edu (C.M.G.); 2 Department of Biostatistics, Yale School of Public Health, New Haven, CT 06511, USA; ralitza.gueorguieva@yale.edu; 3 Child Study Center, Yale University School of Medicine, New Haven, CT 06520, USA; 4 Connecticut Mental Health Center, New Haven, CT 06519, USA; 5 Connecticut Council on Problem Gambling, Wethersfield, CT 06109, USA; 6 Department of Neuroscience, Yale University, New Haven, CT 06520, USA

**Keywords:** food addiction, binge-eating disorder, weighing, obesity, eating disorders, addictive behaviors

## Abstract

Food addiction (FA) has been linked to clinical features in binge-eating disorder (BED) and obesity. A feature of behavioral weight loss (BWL) treatment involves frequent weighing. However, little is known regarding how frequency of self-weighing and related perceptions are associated with BWL outcomes among individuals with BED and obesity stratified by FA status. Participants (*n* = 186) were assessed with the Eating Disorder Examination before and after BWL treatment. Mixed effects models examined FA (presence/absence) before and after (post-treatment and 6- and 12-month follow-up) treatment and associations with frequency of weighing and related perceptions (reactions to weighing, sensitivity to weight gain and shape/weight acceptance). Participants with FA reported more negative reactions to weighing and less acceptance of shape/weight throughout treatment and follow-ups, and both variables were associated with greater disordered eating at follow-ups among participants with FA. Sensitivity to weight gain decreased over time independent of FA status. Frequency of weighing was associated with a greater likelihood of achieving 5% weight loss only among those without FA. Reactions to weighing and sensitivity to weight gain are associated with FA and poorer treatment outcomes in individuals with BED and obesity. Targeting these features may improve BWL outcomes among individuals with BED, obesity and FA.

## 1. Introduction

Changes in the food environment have led to greater exposure to obesogenic foods (i.e., highly palatable, processed, relatively low in cost) and a “toxic food environment” (i.e., the modern food environment encouraging consumption of a diet high in fat and calories) [1]. These changes have been posited to contribute to increased rates of obesity; however, there is considerable debate surrounding whether these types of foods have addictive properties [2,3]. Growing interest and scientific study in the area of food addiction has increased substantially during the past two decades [4], concurrent with the development of self-report measures such as the Yale Food Addiction Scale (YFAS) [5].

The YFAS was developed to standardize assessment of symptoms of addictive-like eating based on diagnostic criteria assessing substance use disorders [5]. Importantly, addictive-like eating behaviors are not currently included within any formal diagnostic category or in any nosological system. However, numerous studies find that food addiction (based on the YFAS) is associated with behaviors/conditions linked to poorer health, including disordered eating, binge-eating disorder (BED) and obesity [6,7,8,9]. Furthermore, prior work suggests that food addiction is strongly associated with poorer body image, including elevated concerns about weight and shape [10,11]. Despite significant work in the past two decades examining the prevalence and clinical correlates of food addiction, few studies have examined the clinical utility of food addiction, and notably there is a scarcity of research investigating individuals with food addiction while receiving evidence-based treatments [12].

There are, however, preliminary findings that food addiction might attenuate weight-loss outcomes among those in behavioral weight loss (BWL) treatment [13]. BWL is an evidence-based treatment for overweight/obesity with goals of modifying problematic eating by establishing patterns of regular eating, restricting caloric consumption and increasing physical activity. Although BWL produces modest weight loss (i.e., 8–10 kg) among individuals with comorbid obesity/BED [14], prior studies have found that greater symptoms of food addiction at baseline were related to less weight loss following participation in a BWL intervention [13], as well as at 12-month follow-up among adults participating in a dietary intervention [15]. However, in other studies, food addiction did not attenuate weight loss [16,17].

In addition to the equivocal findings regarding the predictive significance of food addiction, even less is known regarding how individuals with food addiction perceive and respond to weight-loss interventions, such as BWL. We are unaware of any studies that have prospectively examined changes in behaviors among individuals with and without food addiction during and after treatment. One key component of BWL includes self-monitoring of weight during treatment, and prior work suggests that more frequent or consistent self-weighing is associated with improved weight-loss outcomes [18,19,20,21]. Several prospective studies examining adults during weight-loss treatment found that greater frequency of self-weighing was not associated with adverse psychological outcomes such as binge-eating [22], depression [20,21,23] or other forms of disordered eating, such as compensatory strategies [20,22]. Importantly, however, many of these studies excluded individuals with current or history of eating disorders, and we are unaware of any studies examining self-weighing among those with food addiction.

This present study examined prospectively (during and after BWL treatment) patients with BED with comorbid obesity, with and without food addiction. The first aim was to examine changes in weighing variables, including frequency of self-weighing, reactions to weighing, sensitivity to weight gain and shape/weight acceptance, between groups with and without food addiction during and after BWL treatment. The second aim was to examine associations of weighing variables at post-treatment with binge-eating, disordered eating and weight outcomes following treatment between groups with and without food addiction. We hypothesized that participants with food addiction would endorse greater eating-disorder psychopathology throughout BWL and following treatment compared to those without food addiction. We did not have a priori hypotheses with respect to self-weighing, as no prior studies have assessed the frequency of weighing among those with food addiction.

## 2. Materials and Methods

### 2.1. Participants

Participants were 186 adult (ages 18–65 years) patients with BED and obesity recruited from the community in a large university-based medical health-care center in an urban setting (see [24,25] for detailed description of methods and primary outcomes). All participants were diagnosed with DSM-IV-TR [26] criteria for BED and with obesity (criteria included current BMI ≥30 and ≤50 kg/m^2^). Participants currently using antidepressant medications (a contraindication to the study medications involving sibutramine and orlistat), medications known to influence eating/weight or those with severe psychiatric conditions (e.g., schizophrenia, substance use disorder) or medical problems (e.g., cardiac disease, uncontrolled hypertension, thyroid disease or diabetes) were excluded. Participants were on average 48.38 years old (*SD* = 9.45) and had a mean BMI of 38.88 kg/m^2^ (*SD* = 5.93). The majority of participants were female (71%) and identified as white (84.9%).

### 2.2. Procedures

This study received approval from the University’s Institutional Review Board, and written informed consent was obtained from all participants prior to study procedures. Participants were evaluated by doctoral-level clinicians who were independent assessors with advanced training in eating/weight disorders. Assessors administered the Structured Clinical Interview for DSM-IV Psychiatric Disorders (SCID-I/P; [27]) at baseline to establish a diagnosis of BED and the Eating Disorder Examination Interview (EDE; [28]) to confirm the BED diagnosis at baseline and to comprehensively assess eating-disorder psychopathology at baseline, post-treatment and at 6- and 12-month follow-up assessments. Assessors were blinded to the treatment conditions. Participants completed a battery of self-report questionnaires to characterize associated domains, including food addiction, prior to randomization. Participants were randomly assigned to six-month behavioral weight loss treatment, either an adaptive stepped-care BWL sequential multiple allocation randomized trial (SMART) treatment or “standard” BWL treatment. BWL treatment followed the same protocols in both conditions, which included individual sessions with trained and monitored treatment clinicians following manualized treatment protocols. BWL focuses on making gradual behavioral changes including making moderate increases in physical activity and gradually decreasing caloric consumption. The adaptive SMART stepped-care BWL involved stratifying participants to a different behavioral treatment based on participants’ early response in treatment (i.e., reduction in binge eating). The primary outcomes from this trial have previously been reported (including short- and long-term outcomes), and there were no significant overall differences between the conditions [24,25].

### 2.3. Measures

Weight variables. Following standardized procedures, participant height and weight were measured at participants’ first treatment session using a wall-mounted measure and a large-capacity digital scale (MedWeigh MS-4600 High Capacity BMI Platform Scale). Participants were weighed in street clothing without shoes. Current height and weight at baseline were used to calculate participant BMI (kg/m^2^). Weight was re-measured at post-treatment and six- and twelve-month follow-ups to calculate percent weight change. Weight loss was also examined categorically based on whether participants achieved greater than or equal to 5% weight loss at post-treatment and follow-ups.

The Eating Disorder Examination Interview (EDE; 16th ed; [29]) is a semi-structured, investigator-based interview designed to assess and diagnose eating disorders and eating-disorder psychopathology. Prior psychometric studies of the EDE support its use with BED [30], including with respect to test-retest reliability [31]. The EDE has been shown to differentiate between case and non-cases of eating disorders [32]. In the present study, the EDE—in addition to serving as the primary measure of binge eating and associated eating-disorder psychopathology (i.e., EDE Global Score)—assessed weighing-related variables of primary focus for the current study. These include frequency of self-weighing (henceforth referenced as Weighing), reaction to weekly weighing (henceforth referenced as Reaction), sensitivity to weight gain (henceforth referenced as Sensitivity) and shape/weight acceptance (henceforth referenced as Acceptance). Table 1 describes the study variables and corresponding item from the EDE, which assesses these constructs. Higher scores are indicative of greater pathology.

The EDE also assesses binge-eating episodes, defined as experiencing a subjective sense of loss-of-control while consuming an unusually large amount of food during the past 28 days. Binge-eating episodes were examined as a quantitative variable (number of episodes in past 28 days) and categorically based on binge-eating remission (defined as no binge-eating episodes during the prior 28 days at post-treatment and follow-ups) status. Additionally, the standard EDE global severity (i.e., EDE Global Score) score was calculated, which is comprised the average of four subscales reflecting eating-disorder psychopathology; scores range from 0 to 6, with higher scores reflecting greater severity. It is important to note that none of the weighing variables examined in this study comprise the EDE Global Score. The EDE was administered at pre-treatment, post-treatment and follow-ups.

The Yale Food Addiction Scale (YFAS) [5] is a 25-item self-report measure of food addiction developed in correspondence with substance-dependence criteria from the DSM-IV-TR. The YFAS offers both dimensional (symptom count) and dichotomous (clinical threshold) scoring methods to assess food addiction diagnosis. The YFAS has a one-factor structure and has adequate internal reliability and good convergent validity with measures of problematic eating [5]. For the present study, the dichotomous scoring was used to identify cases with food addiction. The YFAS was administered at pre-treatment, and the pre-treatment assessment was used to determine food addiction status throughout the study period. The Cronbach’s alpha in the present study was 0.88.

### 2.4. Data Analysis

Descriptive statistics were analyzed using SPSS 24.0, and subsequent analyses were conducted in SAS 9.4. To examine changes in weighing variables by food addiction status across the study period, we used mixed effects models with each of the four weighing variables as the response in a separate model, food addiction status (yes/no) and treatment (stepped care vs. BWL) as between-subject factors and time (pre-treatment, post-treatment, 6-month follow-up, 12-month follow-up) as a within-subject factor. Linear mixed models possess several statistical advantages and are considered a robust method for accommodating missing values within longitudinal data. Associations among repeated observations on an individual were modelled using structured variance–covariance matrix with the best structure selected based on the Schwartz Bayesian Criterion (BIC). Transformations were applied prior to analysis in case of non-normality, and residual plots were used to assess the model assumptions. Least square means per food addiction status, treatment and time are shown in all models. Contrasts among least square means were used to explain significant effects. To examine the association of weighing variables at post with dimensional outcomes (i.e., binge-eating frequency, percent weight loss, EDE Global Score) during follow-up, we used mixed effects models with food addiction, time and each weighing variable at post (considered in a separate model) and all their interactions as predictors, controlling for the corresponding outcome at post-treatment as a covariate. Slopes with 95% confidence intervals were estimated when significant effects of weighing variables were observed. For categorical outcomes (i.e., 5% weight loss achieved, binge-eating remission), we fit Generalized Estimating Equations (GEE) models with the same set of predictors as above (without the covariate) and with an exchangeable working correlation structure. Odds ratios with 95% confidence intervals were used to describe significant effects of weighing frequency.

## 3. Results

Of the *n* = 186 participants, 61.3% (*n* = 114) met criteria for food addiction. The average number of food addiction symptoms endorsed by the total sample was 4.77 (*SD* = 1.79) out of the seven total symptoms assessed. Table 2 summarizes the means and standard deviations among food addiction groups for the weighing variables across the assessment timepoints.

### 3.1. Aim 1: Examine Changes in Weighing Variables by Food Addiction Status Over Time

**Figure 1:**
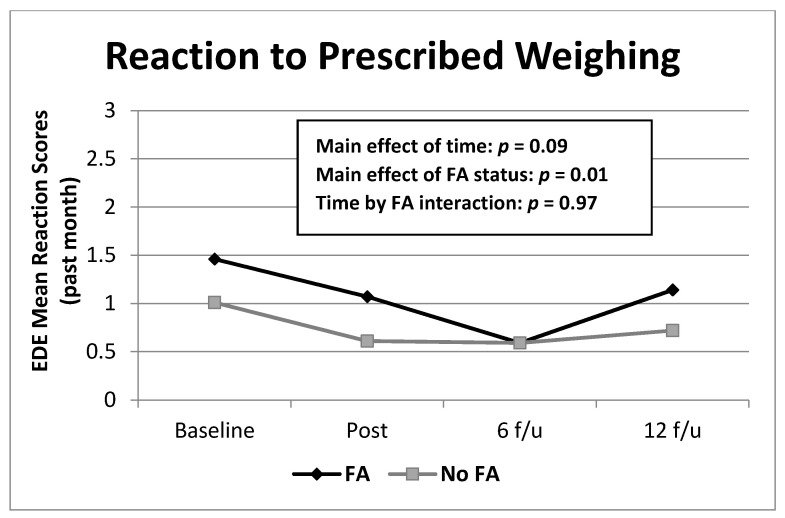
Frequencies of EDE Reaction to prescribed Weighing scores over time by food addiction group. Note: Means were derived from raw data for ease of interpretation. EDE = Eating Disorder Examination; FA = food addiction. Reaction = reaction to prescribed weighing during the past 28 days. Significant main effects were found for food addiction across all time points (*p* < 0.01).

Mixed models analyses of Reaction revealed a significant main effect of food addiction (*F*(1,174) = 6.84, *p* = 0.01). Food addiction was associated with higher Reaction scores across groups and time points (Figure 1).

**Figure 2:**
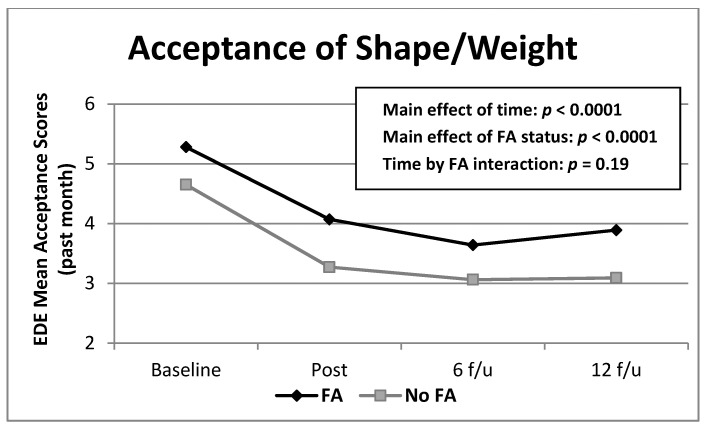
Frequencies of EDE Acceptance of shape/weight scores over time by food addiction group. Note: Means were derived from raw data for ease of interpretation. EDE = Eating Disorder Examination; FA = food addiction. Acceptance = shape/weight acceptance during the past 28 days. Significant main effects were found for food addiction (*p* < 0.0001) and time (*p* < 0.0001). All follow-up scores were significantly different from baseline. Lower scores reflect greater acceptance of shape/weight.

Mixed models analyses of Acceptance revealed significant main effects of food addiction (*F*(1,182) = 16.47 *p* < 0.0001) and time (*F*(3,472) = 37.79, *p* < 0.0001) (Figure 2). Acceptance scores were more pathological for those with food addiction compared to those without. Acceptance scores improved from pre-treatment to the other time points.

**Figure 3:**
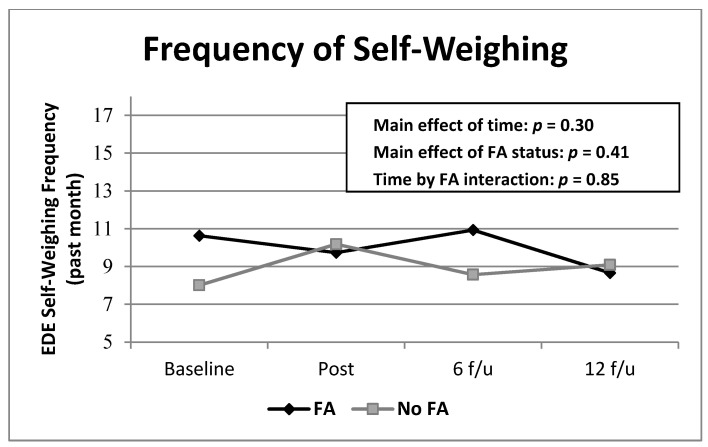
Frequencies of EDE Self-Weighing scores over time by food addiction group. Note. Means were derived from raw data for ease of interpretation. EDE = Eating Disorder Examination; FA = food addiction. All non-significant differences.

Mixed models analyses of Sensitivity revealed a significant improvement from baseline and post-treatment compared to follow-up across conditions *F*(3,465) = 16.94, *p* < 0.0001, but no significant differences between those with and without food addiction *F*(1,178) = 1.59, *p* = 0.21 and no significant interactions. There were no significant effects when examining Weighing scores (see Figure 3).

### 3.2. Aim 2: To Examine the Association of Weighing Variables at Post-Treatment with Binge-Eating, Disordered Eating and Weight Outcomes by Food Addiction Groups

Analyses of binge-eating frequency revealed no significant effects of any of the weighing variables, or interaction with food addiction at follow-ups, when examining binge-eating episodes quantitatively (frequency of episodes) or categorically (remission status).

Mixed model analyses of percent weight loss revealed no significant effects of any of the weighing variables or interactions with food addiction at follow-ups. When examining weight loss categorically (achievement of 5% weight loss or more), however, there was a marginally significant interaction between Weighing at post and food addiction (χ^2^(1) = 3.98, *p* = 0.05) and significant main effects of Weighing at post (χ^2^(1) = 10, *p* = 0.002) and food addiction (χ^2^(1) = 4.73, *p* = 0.03). Increasing Weighing frequency by one unit was associated with almost doubling of the odds of 5% weight loss for subjects without food addiction (OR = 1.95, 95% CI: 1.31, 2.90), whereas the effect was not significant in individuals with food addiction (OR = 1.17, 95% CI: 0.84, 1.61). There were no significant effects on 5% weight loss when examining Reaction, Sensitivity and Acceptance.

Mixed models analyses of the EDE Global Score revealed a significant interaction between Acceptance and food addiction (*F*(1,147) = 4.24, *p* = 0.04) and a significant main effect of Acceptance (*F*(1,148) = 10.27, *p* = 0.002). The slope for the relationship between Acceptance and the EDE Global Score was positive in both groups but significantly steeper for individuals with food addiction. Only the slope in the food addiction group was significantly different from 0 (slope = 0.14, *SE* = 0.03, *p* < 0.0001). There was also a significant interaction between Reaction and food addiction (*F*(1,149) = 4.89, *p* = 0.03). The interaction was due to the slope for the relationship between Reaction and EDE Global Score being slightly positive for those with food addiction and slightly negative for those without food addiction, but neither slope was significantly different from zero. There were no significant effects of Weighing or Sensitivity when examining EDE Global Scores.

## 4. Discussion

This is the first study, to our knowledge, to examine prospectively shape and weight concerns among individuals with and without food addiction participating in weight-loss treatment; more specifically, our study was with patients with BED and comorbid obesity who were subcategorized by food addiction status. Consistent with some of our hypotheses, our findings suggest multiple differences in shape and weight concerns between individuals with and without food addiction, including a stronger negative reaction related to the prospect of weekly weighing, as well as poorer acceptance of shape and weight throughout treatment among those with food addiction. However, no differences in frequency of self-weighing and sensitivity to weight gain were found between groups with and without food addiction.

Furthermore, we found that having a stronger negative reaction to weekly weighing and poorer acceptance of shape/weight following treatment were associated with greater levels of disordered eating following treatment among those with food addiction but were not related to binge-eating or weight-loss treatment outcomes. Sensitivity to weight gain was also unrelated to treatment outcomes and decreased over time across participants. Last, frequency of self-weighing was relatively stable over time and was not related to treatment outcomes (i.e., binge-eating, percent weight loss, disordered eating). However, greater frequency of weighing following treatment was related to a greater likelihood of achieving 5% weight loss following BWL treatment among those without food addiction.

The first aim of this study was to examine changes in weighing variables, including frequency of self-weighing, reactions to weighing, sensitivity to weight and shape/weight acceptance before and after BWL treatment between groups with and without food addiction. There were no significant differences in frequencies of self-weighing and sensitivity to weight gain when comparing those with and without food addiction and no significant changes after BWL treatment. However, participants categorized with food addiction endorsed a stronger negative reaction to weekly weighing and poorer acceptance of shape/weight over time relative to those without food addiction. It is important to note that although the food addiction group endorsed more pathological scores related to the prospect of weekly weighing, both groups endorsed subclinical scores (i.e., scores ≤4) throughout the assessment period. Shape/weight acceptance scores, however, were clinically elevated among both groups prior to starting treatment and remained in the clinical range for those with food addiction at the post-treatment assessment. Taken together, these longitudinal findings extend prior cross-sectional work suggesting that the combination of BED and food addiction are associated with elevated eating-disorder psychopathology [10,11,33] and suggest that food addiction, if present in patients with BED, might warrant additional clinical focus during BWL treatment.

The second aim was to examine whether changes in weighing variables (i.e., self-weighing, reaction to weekly weighing, sensitivity to weight gain and shape/weight acceptance) following treatment were associated with treatment outcomes among those with and without food addiction following BWL treatment. Prior work examining self-weighing within adult samples with overweight highlight the significant benefits of consistent self-weighing on weight loss outcomes [18,19,20,21], yet individuals with binge-eating or those with a history of eating disorders are often excluded from weight-loss studies. The present findings suggest that frequency of weighing was not significantly related to adverse treatment outcomes, including binge-eating, percent weight loss and eating-disorder psychopathology in a clinical sample of individuals with obesity and BED. Our prospective findings add to the growing literature suggesting that self-weighing in patients with BED with comorbid obesity might not have negative effects [34], and this seems to be the case for those with and without food addiction. Importantly, studies examining individuals with other eating disorders characterized by highly restrictive eating behaviors (i.e., anorexia nervosa, bulimia nervosa) or young adult women and girls, however, have found that more frequent self-weighing is associated with greater eating-disorder psychopathology [34,35,36,37]. The present study also found that individuals without food addiction who weighed more often were significantly more likely to achieve 5% weight loss, suggesting some benefit to regularly weighing. However, this result was observed only among those without food addiction. Participants were self-weighing, on average, ten times in a 28-day period, which approximates to weighing 2.5 times per week. An important direction for future research is to determine the threshold at which more frequent self-weighing may become maladaptive in those with BED [38]. Taken together, our findings highlight the benefits of self-weighing to promote weight loss in individuals with BED and obesity without food addiction.

Additionally, we found that endorsing a stronger negative reaction to weekly weighing and poorer acceptance of shape/weight were associated with greater disordered eating following treatment among those with food addiction and BED. Participants with co-occurring food addiction and BED did not self-weigh more often than those with BED without food addiction, yet consistently endorsed a stronger negative reaction to the prospect of weekly weighing. These findings have possible implications for treatments such as BWL and cognitive behavioral therapy (CBT), an evidence-based treatment for BED [39,40]. CBT for BED recommends limiting self-weighing to weekly, as opposed to daily self-weighing, which is common for some BWL treatments. Thus, our findings highlight possible areas for assessment among participants with food addiction, who endorsed a strong reaction to the prospect of weekly weighing. Assessing reactions and perceptions of weekly weighing in patients with food addiction may be helpful to identify potential barriers to weighing interventions in treatments such as BWL and CBT. Additionally, our findings suggest that less acceptance of shape/weight was associated with greater eating-disorder psychopathology in this subgroup. Future studies testing in patients with food addiction the efficacy of CBT, which provides durable and significant improvement in cognitive symptoms for individuals with BED [41], are warranted. Taken together, our findings suggest that certain body image concerns are a negative prognostic indicator among those with food addiction, which highlight important targets for treatment in this subgroup of patients.

There are several limitations to the present study to highlight. Although self-weighing was assessed using a semi-structured diagnostic interview of eating-disorder psychopathology, the frequency of self-weighing is based on participants retrospective report. Future work should assess self-weighing using electronic scales to determine objectively assessed self-weighing. Participants in this study were predominately female and white; thus, generalizability is limited. Future studies should evaluate these outcomes in more diverse samples including larger samples with more male participants. Future studies should also examine these outcomes using the more recent version of the self-report assessment of food addiction (i.e., YFAS 2.0), which corresponds to the DSM-5 definitions of substance use disorders, as the YFAS 2.0 was not yet developed when this study was conducted.

## 5. Conclusions

In summary, results of the present investigation provide evidence that self-weighing among individuals with BED with comorbid obesity with and without food addiction is not associated with poorer eating-disorder psychopathology or weight outcomes following BWL treatment. Frequency of self-weighing was associated with a marginally greater likelihood of achieving 5% weight loss, but only in those without food addiction. Our findings suggest that individuals categorized with food addiction reported a stronger negative reaction to weekly weighing and poorer acceptance of shape/weight, which were prospectively associated with greater eating-disorder psychopathology but were not related to weight loss outcomes and binge-eating frequency or remission. Clinicians should assess and consider body image concerns in treatment conceptualization and delivery in patients with BED comorbid with obesity who also report food addiction.

## Figures and Tables

**Table 1:** Weighing and body image variables prospectively examined.

Study Variables	EDE Item	Frequency/Rating
Weighing	“Over the past 4 weeks how often have you weighed yourself?”	Number of times weighed in past 28 days
Sensitivity	“Over the past 4 weeks what amount of weight gain, over a period of 1 week, would have definitely upset you?”	7-point Likert scale based on the number of pounds or kilograms that would generate a marked negative reaction (0 = 7 lbs or 3.5 kg or more to 6 = 1 lb or 0.5 kg).
Reaction	“Over the past 4 weeks how would you have felt if you had been asked to weigh yourself once each week for the subsequent 4 weeks … just once a week; no more often and no less often?”	7-point Likert score ranging from 0 = no reaction to 6 = marked reaction (pronounced reaction which would affect other aspects of the subject’s life).
Acceptance	“Over the past 4 weeks, to what extent have you been able to accept your shape and weight—see them as simply being the way you are?”	7-point Likert scale ranging from 0 = complete acceptance to 6 = no acceptance.

Note: EDE = Eating Disorder Examination Interview. Items obtained from the EDE Interview [29].

**Table 2:** Means and standard deviations of weighing variables by Yale Food Addiction Scale Group.

	Pre-Treatment		Post-Treatment		6-Month Follow-Up		12-Month Follow-Up		
	FA (*n* = 114)	No FA (*n* = 72)	FA (*n* = 100)	No FA (*n* = 66)	FA (*n* = 114)	No FA (*n* = 72)	FA (*n* = 91)	No FA (*n* = 63)	Sig.
Weighing	10.63 (15.00)	8.01 (11.10)	9.73 (11.98)	10.18 (17.01)	10.93 (19.32)	8.57 (10.35)	8.66 (10.42)	9.09 (11.63)	ns
Sensitivity	4.31 (1.73)	4.40 (1.63)	4.17 (1.69)	3.92 (1.45)	3.45 (1.80)	3.17 (1.81)	3.68 (1.89)	3.19 (1.62)	0.0001
Reaction	1.46 (1.88)	1.01 (1.64)	1.07 (1.77)	0.61 (1.39)	0.59 (1.41)	0.59 (1.41)	1.14 (1.81)	0.72 (1.59)	0.01
Acceptance	5.28 (1.20)	4.65 (1.20)	4.07 (1.73)	3.27 (1.64)	3.64 (1.87)	3.06 (1.74)	3.89 (1.85)	3.09 (1.67)	0.0001

Note: Means were derived from raw data for ease of interpretation. FA = food addiction. ns = non-significant effects. Weighing = frequency of self-weighing during the past 28 days. Sensitivity = sensitivity to weight gain during the past 28 days. Follow-up scores all significantly different from pre-treatment. Reaction = reaction to prescribed weighing during the past 28 days. Significant main effects for time and FA status. Acceptance = shape/weight acceptance during the past 28 days. Significant main effects for time and FA status.

## Data Availability

De-identified data will be provided in response to reasonable written request to achieve specified goals in an IRB-approved written proposal. These data are not published available at this time as they continue to be analyzed as part of on-going grant-funded projects.
